# Old Colony Dental Association

**Published:** 1867-06

**Authors:** L. W. Puffer


### OLD COLONY DENTAL ASSOCIATION.

In accordance with a call issued by the Dentists of South-Eastern Massachusetts, who were present at the recent session of the United States Dental Association, in Boston, a convention of Dentists was held at Middleboro, on the 15th of August, 1866, to consider the subject of forming an Association in this section of the State.

Dr. D. S. Dickerman, of Taunton, was chosen President, *pro tem.*, and Dr. C. E. Williams, of Fall River, Secretary. Voted to organize a society to be called the "Old Colony Dental Association." A constitution was adopted, and the following Officers elected:—President, D. S. Dickerman, D. D. S., of Taunton; Vice-President, ——— ; Recording Secretary, George R. Whitney, of North Bridgewater; Corresponding Secretary, Loring W. Puffer, of North Bridgewater; Librarian and Treasurer, Julius Thompson, of Taunton.

*Executive Committee.*

N. C. Fowler,

C. G. Davis,

Julius Thompson,

C. E. Williams,

L. W. Puffer,

The first meeting of the Association was held at Bridgewater, Sept. 10th. An essay on alveolar abscess was read by Dr. D. S. Dickerman, of Taunton. Clinic by Dr. N. C. Fowler, of Yarmouthport. The second meeting was held at Taunton, Nov. 12th; J. C. Parkard, of North Bridgewater, J. Q. Dickerman, and James Utley, of Taunton, were elected members of this Association. Clinic by Dr. Thompson, of Taunton; Essay by Dr. C. G. Davis, of New Bedford, on the comparative value of Anæsthetics. The third meeting of the Association was held at the Parke's House, New Bedford, January 14th, 1867. Clinics by Dr. D. S. Dickerman, of Taunton, and C. G. Davis, of New Bedford. Dr. J. Q. Dickerman, of Taunton, explained Dr. Hurd's method of taking inpresssions (in flat mouths?). The fourth meeting was held at Taunton, March 12th, 1867. Dr. Thompson read an essay on the extraction of teeth; a revised Constitution, By-Laws and Code of Ethics were adopted, and a Committee appointed to have the same printed. Drs. D. S. Dickerman, of Taunton, C. G. Davis, of New Bedford, and L. W. Puffer, of North Bridgewater, were elected delegates to represent this Association, at the next meeting of the American Dental Association. Hereafter the meetings will be quarterly. The next meeting will be held on April 10th, at North Bridgewater. Sessions day and evening. The profession are invited. Essay by Dr. Fowler, of Yarmouthport. Subject for discussion—Inflammation of the Gums.

L. W. PUFFER, *Cor. Sec'y.*

